# A new mutant genetic resource for tomato crop improvement by TILLING technology

**DOI:** 10.1186/1756-0500-3-69

**Published:** 2010-03-12

**Authors:** Silvia Minoia, Angelo Petrozza, Olimpia D'Onofrio, Florence Piron, Giuseppina Mosca, Giovanni Sozio, Francesco Cellini, Abdelhafid Bendahmane, Filomena Carriero

**Affiliations:** 1 Metapontum Agrobios, SS Jonica 106 Km 448.2, 75010 Metaponto (MT), Italy; 2 Unité de Recherche en Génomique Végétale, UMR INRA-CNRS, Rue Gaston Crémieux, 91057 Evry Cedex, France; 3 ENEA, Casaccia Research Center, PO Box 2400 Roma 00100AD, Italy

## Abstract

**Background:**

In the last decade, the availability of gene sequences of many plant species, including tomato, has encouraged the development of strategies that do not rely on genetic transformation techniques (GMOs) for imparting desired traits in crops. One of these new emerging technology is TILLING (Targeting Induced Local Lesions In Genomes), a reverse genetics tool, which is proving to be very valuable in creating new traits in different crop species.

**Results:**

To apply TILLING to tomato, a new mutant collection was generated in the genetic background of the processing tomato cultivar Red Setter by treating seeds with two different ethylemethane sulfonate doses (0.7% and 1%). An associated phenotype database, LycoTILL, was developed and a TILLING platform was also established. The interactive and evolving database is available online to the community for phenotypic alteration inquiries. To validate the Red Setter TILLING platform, induced point mutations were searched in 7 tomato genes with the mismatch-specific ENDO1 nuclease. In total 9.5 kb of tomato genome were screened and 66 nucleotide substitutions were identified. The overall mutation density was estimated and it resulted to be 1/322 kb and 1/574 kb for the 1% EMS and 0.7% EMS treatment respectively.

**Conclusions:**

The mutation density estimated in our collection and its comparison with other TILLING populations demonstrate that the Red Setter genetic resource is suitable for use in high-throughput mutation discovery. The Red Setter TILLING platform is open to the research community and is publicly available via web for requesting mutation screening services.

## Background

Tomato (*Solanum lycopersicum*) is one of the most important vegetable plants in the world. Its fruits are end products both for the fresh market and food processing industry. Tomato presents a relatively small genome highly syntenic to others economically important Solanaceae species and was selected as a reference species for sequencing a Solanaceae genome. In addition to the availability of a number of genomic resources, including transcriptome [1-3] and metabolome [4], large collections of genetic resources are available to dissect the biochemical and the metabolic pathways in tomato [5]. Large EMS and fast neutron mutant collections, in the background of M82 tomato cultivar, have been generated and more then 3,000 phenotype alterations catalogued [6]. An EMS-induced mutation library of the miniature dwarf tomato cultivar Micro-Tom has also been produced and this constitutes another resource for tomato genetic studies [7].

In recent years, the genome sequencing program of many plant species [8-10], including tomato [11] has led to the availability of a large number of gene sequences in public databases which subsequently has encouraged the development of reverse genetics approaches. T-DNA and transposon insertional mutagenesis have been exploited to inactivate genes in tomato [12,13]. However, unless a high-throughput tomato transformation protocol is developed, systematic functional analysis of tomato genes with these approaches is not realistic. In recent years, TILLING (Targeting Induced Local Lesions IN Genomes) [14,15] a new emerging technology that doesn't rely on genetic transformation techniques, allows systematic functional genomic studies. The only prerequisite for its application is the knowledge of the gene nucleotide sequences. TILLING is a reverse genetic strategy that utilises chemical mutagenesis for inducing variability and sensitive molecular screenings to identify point mutations responsible for phenotype alteration. The strength and potency of this reverse genetic strategy has been validated by its successful application in both plants (*Arabidopsis thaliana *[16-18], pea [19], wheat [20], rice [21,22], barley [23], maize [24], soybean [25], *Lotus japonicus *[26], sorghum [27], tomato [28,29]) and animals (zebrafish [30], drosophila [31]).

In the present paper we report the construction of a high-quality tomato genetic mutant reference collection which could be used for both forward and reverse genetic studies. We have developed such a population by mutagenizing the processing tomato variety Red Setter with EMS and establishing an associated phenotype database, LycoTILL, and a TILLING platform. The database also serves as a portal for users to request materials or TILLING experiments.

## Results

### Generation of the mutant collection

Red Setter is a processing tomato variety that completes its reproductive cycle within 110 days, it is a high productive variety and its architecture permits mechanical harvesting. In order to optimize the EMS mutagenesis, we first conducted a 'kill-curve' analysis, using a range of doses from 0.3-1.5% EMS. Two EMS doses were then chosen to generate the mutant collection. The first mutagen treatment was performed by incubating about 11,000 seeds with 0.7% EMS that caused 20% reduction in seed germination (LD_20_) with respect to untreated control seeds. The second mutant tomato population was produced by treating 12,000 seeds with 1% EMS (LD_49_). Out of the 23,000 treated seeds, 13,000 seedlings were grown to fruit maturity in controlled conditions and M2 seeds were collected from individual M1 plants from different plant internodes. In total we collected 6,667 distinct M2 seed stocks, among which 4,741 and 1,926 M2 seed stocks were obtained with 0.7% and 1% EMS treatment respectively. For the production of M3 seed stocks, three seeds per M2 family were sown in nursery, grown to fruit maturity in open field and M3 seeds harvested from individual M2 plants. In total we collected 5,508 M3 seed stocks (Table 1) as 1,159 M2 families out of 6,667 M2 families didn't produce M3 seeds. Specifically there were 585 M2 families (12.33%) generated from the 0.7% mutagenesis experiment and 574 M2 families (29.8%) generated from the 1% EMS treatment.

**Table 1:** Summary of the Red Setter tomato mutant collection development

EMS Concentration	Mutagenized Seeds (No)	Transplanted M1 plants	M2 seed Families	M3 seed Families
**0.7% _(LD 20)_**	11,000	8,500	4,741	4,156
**1% _(LD 49)_**	12,000	4,500	1,926	1,352
Total	**23,000**	**13,000**	**6,667**	**5,508**

EMS concentrations, number of seeds treated with the mutagen, number of M1 plants and collected M2 and M3 seed families are shown.

### M2 plant phenotyping

Three plants per M2 family of Red Setter mutant population were scored for visual phenotype alteration at key developmental stages, from germination until fruit maturation. The data collected from individual plants were organized in 17 classes and 51 subclasses of phenotypes. The vocabulary used to describe the phenotypes was derived from the plant phenotype ontology and from previous investigation of systematic phenotyping of the mutant tomato collection [6]. We also introduced three new classes of phenotype alterations, the *cotyledon*, the *fruit number *and the *seed germination into fruit*. The *cotyledon *class describes mutants showing alterations in the number, color and morphology of the cotyledons. The *fruit number *class describes mutants affected in the fruit yield and it contains three subclasses, few, many or absence of fruits. By adding the *fruit number *class and the subclass "absent" we distinguished the plant sterility due to the absence of fruits from those caused by the seedless fruits. The class of phenotype *seed germination into fruit *describes mutants having pre-germinated seeds still in the fruit. This phenotype is presumed to result from an altered fruit flesh pH or by a hormonal imbalance [32,33]. The complete list of the vocabulary used and the number of lines found in each major phenotype category are shown in Table 2.

**Table 2:** List of phenotype classes and subclasses

	Class	Subclass	No. of plants
1	Seed	No germination	3,904
		Seedling lethality	1,674
2	Cotyledons	Colour	264
		Number	82
		Morphology	17
		Size	2
		Other cotyledon development	3
3	Plant size	Small plant	303
		Large plant	4
4	Plant habit	Aborted growth	81
		Branching	109
		Internode length	16
		Other plant habit	174
5	Leaf morphology	Leaf complexity	27
		Leaf size	98
		Leaf texture	16
		Leaf width	12
		Other leaf development	188
6	Leaf colour	Dark green leaf	19
		Dull green/grey leaf	19
		Purple leaf	17
		Variegation	20
		White leaf	4
		Yellow leaf	28
		Yellow-green leaf	79
7	Flowering	Late flowering	142
8	Inflorescence	Inflorescence structure	28
9	Flower morphology	Flower homeotic mutation	5
		Flower organ size	10
		Flower organ width	12
		Other flower morphology	4
10	Flower colour	Pale yellow flower	13
		White flower	5
11	Fruit size	Large fruit	212
		Small fruit	192
12	Fruit morphology	Long fruit	43
		Other fruit morphology	2
		Rounded fruit	4
13	Fruit colour	Dark red fruit	0
		Green fruit	0
		Orange fruit	0
		Yellow fruit	3
14	Fruit number	Absent	1,073
		Few	1,393
		Many	64
15	Sterility	Partial sterility	2,125
		Total sterility	576
16	Seed germination into fruit	Seed germination into fruit	208
17	Disease and stress response	Necrosis	48
		Wilting	80
		Other disease response	0

Number of plants bearing a specific mutant phenotype. Since a single plant may also be recorded more than once, if it was scored for more than one phenotype, the numbers are not additive.

The data reported in this table refer to the phase of M2 population development.

**Figure 1:**
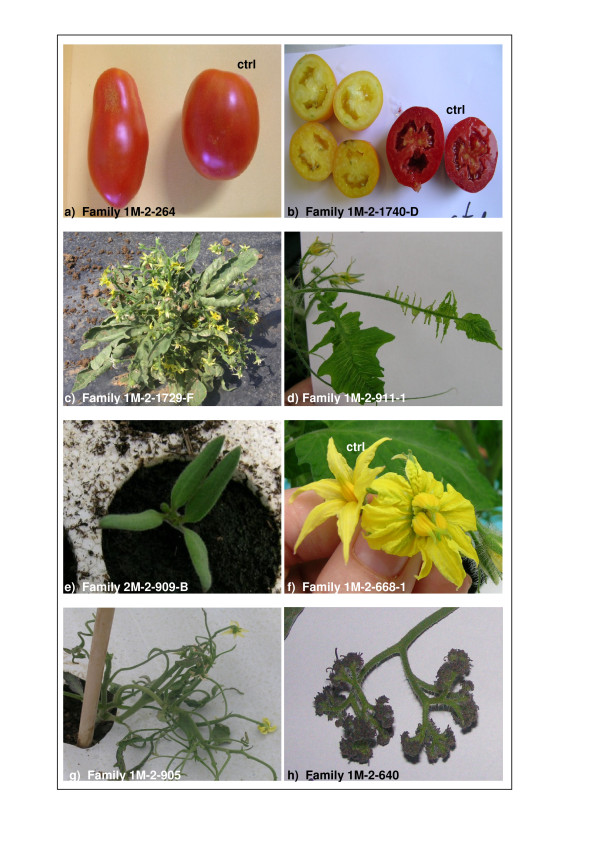
**Examples of tomato mutant phenotypes**. Mutations affecting: a) fruit morphology, b) fruit colour, c) plant habit, d) leaf morphology, e) cotyledon number, f) flower morphology, g) plant habit, h) inflorescence structure.

39% of tomato M2 plants showed at least one visual mutant trait and among these lines 37% displayed multiple phenotypes that fall into more than one major class of phenotypes. The most commonly observed phenotypes are related to the cotyledons (368), the leaf morphology (341), the habit (380) and the plant size (307) classes. In Figure 1 examples of tomato mutant traits are shown.

### LycoTILL database

In order to manage and integrate the recorded phenotypic data, we implemented the database LycoTILL. LycoTILL was developed according to a relational database system, interconnecting three main modules: lines, class and subclass of phenotypes. The database interrogation can be done according to the phenotypic catalog, previously reported, or by plant code number (plant name) or family name. The result displays all the collected phenotypic information as well as photos of the mutant lines. LycoTILL, that is an evolving database, is publicly accessible through the web interface: http://www.agrobios.it/tilling/index.html[34].

### Mutation discovery in Red Setter mutant collection

To set up the tomato TILLING platform, DNA samples were prepared from 5,221 M3 families corresponding to 3,924 and 1,297 families obtained by treatment with 0.7% and 1% EMS respectively. The selection of M3 families was based on the M3 seed abundance, 287 families were discarded due to their low seed set. DNA samples were organized in pools of 8 M3 families. To validate the 0.7% and the 1% EMS Red Setter TILLING platforms and to estimate the mutation density of the populations, we chose seven genes involved in fruit quality traits. In particular we analysed ripening-inhibitor (*RIN*) and green ripe (*Gr*) genes involved in the ripening of tomato fruit, rab11 GTPase (*Rab11a*) and expansin 1 (*Exp1*) genes related to the tomato softening control, polygalacturonase (*PG*) gene involved in the cell wall hydrolysis, and lycopene beta cyclase (*Lcy-b*) and lycopene epsilon cyclase (*Lcy-e*) involved in the carotenoid biosynthesis pathway.

The CODDLE program (Codons Optimized to Discover Deleterious Lesions [35,36]) combined with the PRIMER3 tool [37] was used to define the best amplicon for TILLING analysis. Mutations were detected in the amplified targets using the mismatch-specific endonuclease ENDO1 as previously described [19,38].

In total 9.5 kb of tomato genome were screened and 66 induced point mutations were identified (Table 3) of which 41 and 25 mutations were derived from the 0.7% and 1% EMS treated populations respectively. As expected for EMS mutagenesis, single nucleotide substitutions were identified both in coding and non-coding regions [17]. Among the exonic identified mutations, 37.6% were silent and 62.4% were missense mutations while no stop codon type of mutations was found. Using the SIFT programme (Sorting Intolerant From Tolerant [39,40]), we analysed the putative impact of the missense mutations on the function of the tilled genes and 57.14% of the missense mutations were predicted deleterious for the protein's activity.

**Table 3:** Mutation density in 0.7% EMS and 1% EMS Red Setter populations

Target gene		No. of screened M3 families		No. of identified mutations		Overall mutation density	
Name	Amplicon size (kb)	0.7% EMS	1% EMS	0.7% EMS	1% EMS	0.7% EMS	1% EMS
***Rab11a***	0.407	1,373	713	1	3	1/559 kb	1/97 kb
***PG***	2.587	2,791	963	7	2	1/1031 kb	1/1246 kb
***Exp1***	1.025	3,885	1,284	14	6	1/284 kb	1/219 kb
***RIN***	1.331	3,885	1,284	4	8	1/1293 kb	1/214 kb
***Gr***	1.409	3,885	1,284	5	3	1/1095 kb	1/603 kb
***Lcy-b***	1.274	3,801	1,252	4	3	1/1211 kb	1/532 kb
***Lcy-e***	1.414	3,630	1,185	6	0	1/855 kb	-
Total/mean	**9.447**			**41**	**25**	**1/574 kb**	**1/322 kb**

The accession numbers of the analyzed seven target genes are the following: *Rab11a *[GenBank:AJ245570], *PG *[GenBank:M37304], *Exp1 *[GenBank:AF548376], *RIN *[GenBank:AF448522], *Gr *[GenBank:DQ372897], *Lcy-b *[GenBank:CQ788383], *Lcy-e *[GenBank:Y14387]. The number of screened M3 families, the number of identified mutations and the overall mutation density, estimated as described in Methods, are reported both for 0.7% and 1% EMS Red Setter populations.

We calculated the mutation density in the seven targeted genes (Table 3) according to Dalmais *et al*. [19]and Greene *et al*. [17]. We estimated the mutation density at 1 mutation/322 kb in the 1% EMS and 1 mutation/574 kb in the 0.7% EMS Red Setter population.

## Discussion

The optimization of mutagenesis is a critical parameter in establishing a good mutant collection for forward and reverse genetic studies. In order to balance maximum mutation density with an acceptable plant survival rate we decided to utilise two different doses of EMS, 0.7% and 1%. A strict correlation was observed between the EMS doses and the toxicity, the mutation density obtained and the frequency of phenotype alterations. At 1% EMS the plant fertility rate was 41% less than the plant treated with 0.7%. In contrast, the 1% EMS yielded 1.78 fold more mutations per genome than 0.7% treated plant. At the phenotype level, 60% of the mutant phenotypes scored in the M2 generation were derived from the 1% EMS treated seeds.

In the TILLING screens we analysed seven genes and discovered a total of 66 induced point mutations. The spectrum of expected mutations in an EMS-treated population is essentially GC/AT transition because of the frequent alkylation of guanine residues by EMS [41]. In *Arabidopsis*, maize, wheat and pea, more than 99% of identified mutations are GC/AT transition [17,19,20,24]. In our mutant Red Setter populations the percentage of observed GC/AT transition was 60% in the 0.7% EMS population and only 28.6% in the 1% EMS population. We also identified GC/TA, AT/TA, AT/CG, GC/CG and AT/GC transversions (Table 4). The spectrum of observed nucleotide changes in the 0.7% EMS-treated tomato population is similar to the spectrum of mutations observed in the reverse TILLING screens of rice and barley for which transitions in the range of 70% are reported [21,23]. In contrast, the mutational spectrum of the 1% EMS population is different (AT/CG, GC/CG, see Table 4).

**Table 4:** Spectrum of mutations identified in Red Setter populations and their comparison to other organisms

Mutation		Tomato Red Setter		**Barley **[23]	**Rice **[21]
Type	Change	0.7% EMS	1% EMS		
Transition	GC/AT	**60.0**	**28.6**	70.0	70.0
Transversion	GC/TA	**6.7**	**14.3**	10.0	4.0
	AT/TA	**20.0**	**14.3**	10.0	15.0
	AT/GC	**13.3**	**14.3**	10.0	11.0
	AT/CG	**0**	**14.3**	0	0
	GC/CG	**0**	**14.3**	0	0
Total (%)		100	100	100	100

Distribution of the identified mutations in the different classes of nucleotide changes. In addition to the Red Setter mutant population data, reported as percentage values, the mutation spectrum of barley and rice are also shown.

In order to rule out the probability that natural polymorphisms, introduced through pollen or seed contamination, could be responsible for the non-GC/AT changes observed in our mutant populations, we analysed the natural sequence variation of the tilled genes using BLAST analysis [Additional file 1: Supplemental Figure S1] and EcoTILLING [42] of 150 tomato varieties among which 45 were Italian varieties (unpublished data). These analyses revealed that the nucleotide changes identified by TILLING were present neither in the available gene bank sequences nor in the screened tomato varieties. Based on this, we concluded that the non-GC/AT changes discovered in the TILLING screens do not result from cross pollination, but are new allelic variants generated by the mutagen action. This conclusion is also consistent with the non recovery of non-GC/AT changes in multiple genes in the same individual as reported for the Seattle Arabidopsis population, where rare contaminants were observed to introduce polymorphisms in more than one gene in the same plant [17].

Based on this we speculate that tomato might differ from other plant species in its mutagenic response to EMS doses. Moreover, we think that the choice of ENDO1 enzyme was fundamental in the detection of all types of changes that we observed in our mutant populations. For its high specificity in recognizing mismatches at the same rate [38] we could identify mutations never found in other plant species and with a higher frequency.

The TILLING screening performed on seven tomato genes permitted the calculation of the mutation density in the two mutant Red Setter populations. We estimated the mutation density at 1 mutation/322 kb in the 1% EMS and 1 mutation/574 kb in the 0.7% EMS Red Setter population. The mutation densities calculated in the 1% and 0.7% EMS Red Setter populations are 2.3 and 1.2 times respectively higher than one mutation every 737 kb reported by Gady et al[29] in the 1% EMS TPAADASU tomato population.

This comparison demonstrate that our populations have a higher number of mutations respect to those so far available and published for tomato. The high mutation density of our populations, especially for the 1% EMS one, increases the size of allelic series that can be obtain and reduces the population size that needs to be screened.

Comparing the mutation densities estimated in the 1% and 0.7% EMS Red Setter populations with those described in other plant species results that they are 1.9 and 3.4 times respectively lower than one mutation per 170 kb reported previously for *Arabidopsis *[17] but their average (1/448 kb) is similar to those reported for maize (1/500 kb) [24] and rice (1/500 kb) [22] and 2.2 fold higher than one mutation per Mb found in barley by Caldwell *et al*. [23].

So far higher mutation densities were observed only in tetraploid wheat (1/40 kb) and hexaploid wheat (1/24 kb) [20]. It's likely that the polyploidy nature of their genomes helps in withstanding the mutagen action and consequently higher mutation frequencies can be obtained.

A mutant population is considered saturated with at least a single "hit" in every gene [6]. In the Red Setter TILLING platform more than one mutation was identified per gene analysed. We can therefore conclude that our mutant populations are sufficiently saturated. Furthermore by comparing other plant species used in public TILLING projects we can also affirm that our populations are suitable for use in high-throughput mutation discovery.

## Conclusions

We have developed a new genetic resource in the tomato Red Setter genetic background by means of EMS mutagenesis. The mutant collection is organized as such that it could be used for both forward (EMS saturated mutant collection and the associated phenotypic database) and reverse (high-throughput TILLING platform) genetics in tomato, for both basic science or crop improvement.

The Red Setter TILLING platform is open to the scientific community to request TILLING screenings in genes of interest and to obtain material.

These services can be requested *via *database that also serves as portal for user need. In addition to our platform, at present, other tomato TILLING platforms are publicly accessible via web for requesting TILLING services (http://urgv.evry.inra.fr/UTILLdb and http://tilling.ucdavis.edu/index.php/TomatoTilling). All the available tomato TILLING platforms, including the Red Setter one, utilise mutant collections generated in different genetic backgrounds and with different EMS doses which increase the chance of obtaining a larger spectrum of alleles. Thus, it is of interest for the scientific community to have different tomato TILLING resources for the possibility of identifying a greater number of mutations of interest.

## Methods

### EMS mutagenesis

Tomato seeds (cv Red Setter) were treated with two different concentrations (0.7% and 1%) of the chemical mutagen EMS (ethylmethane sulfonate) for 18 h at RT with gentle shaking. The seeds were then thoroughly washed, dried and sown in compost in 96 well seed trays which allowed an accurate determination of germination frequency.

Control seeds, those not exposed to EMS treatment, were treated in the same manner.

### Plant material

*M2 seeds*: for the M2 seed production, M1 plants were grown according to standard tomato agronomic practice and at the end of the fruit-ripening phase, M2 seeds were collected from individual M1 plants and kept separate.

*M3 seeds*: 3 seeds belonging to each mutant M2 family were sown in 96 well seed trays and the corresponding seedlings transplanted in open field. M3 seeds were collected from single M2 plants.

### M2 plant phenotyping and data collection tools

Phenotype scoring was performed at different developmental stages from seed germination through fruit ripening and seed harvest. Each mutant candidate was characterized according to 17 classes and 51 subclasses which are reported in Table 2. The selection of classes and subclasses was for the most part carried out on the basis of the phenotypic catalog reported by Menda *et al*. [6].

Data were collected using a hand-held Asus MyPal 730w while pictures were taken by using the Nikon Coolpix 4500 digital camera.

### Database construction

The phenotype database was developed using MySQL [43] as a relational database system.

### DNA extraction and sample pooling

For each M3 family, the genomic DNA was extracted from four young leaves collected from four different plants of the same family. The leaf samples were collected in 96-well plates and the DNA was isolated by using DNeasy 96 Plant Kit (Quiagen, Hilden, Germany). The quantification of extracted DNA was carried out on a 0.8% agarose gel using λ DNA (Invitrogen, Carlsbad, CA, USA) as a concentration reference. Genomic DNA samples were then diluted tenfold and pooled eightfold to obtain the working material.

### PCR amplification, mutation detection and validation

PCR amplification was based on nested-PCR and was carried out using two couples of target-specific primers. 4 ng of pooled genomic DNA was used for the first PCR and forward-strand primers and reverse-strand primers 5'-end labelled with IRDye 700 and IRDye 800 dye (LI-COR^®^, Lincoln, NE, USA) respectively were employed for the second PCR [19].

Mutation detection was performed as previously described [38]. Electrophoresis were performed on a LI-COR 4300 (LI-COR^®^, Lincoln, NE, USA) and gel images were analysed using Adobe Photoshop software (Adobe Systems Inc., San José, CA, USA).

After discovery, mutations were validated by sequence analysis.

The mutation frequency for each amplicon was calculated as previously described [19]. For the average mutation frequency we have summed the sizes of all amplicons and we have divided by the total number of identified mutants. The data were multiplied for 0.75 in order to eliminate the missing evaluation due to the presence of one-fourth wild-type alleles in the 1:2:1 Mendelian segregation in M3 generation [17].

## Abbreviations

BLAST: Basic Local Alignment Search Tool; CODDLE: Codons Optimized to Discover Deleterious Lesions; ctrl: control; EMS: ethylmethane sulfonate; LD: Lethal Dose; NCBI: National Center for Biotechnology Information; RT: Room Temperature; SIFT: Sorting Intolerant From Tolerant; TILLING: Targeting Induced Local Lesions IN Genomes.

## Competing interests

The authors declare that they have no competing interests.

## Authors' contributions

FCa planned and headed the development of the mutant populations. AP and GS took care and visually phenotyped the mutant populations; OD set up the LycoTILL database; SM extracted the DNA; SM, FP and GM did TILLING screens and analysis; AB supervised the TILLING platform set up in Evry; FCe and FCa co-directed the TILLING project in M. Agrobios; FCa and SM were responsible for drafting and revising the manuscript with contributions from co-authors.

All authors read and approved the final manuscript.

## Supplementary Material

Additional file 1 **Nucleotide alignment**. Additional data file 1 is a figure showing a comparison analysis of a tilled 240 bp region of *Expansin1 *gene. This analysis shows that the identified induced point mutations are not part of natural variability. Click here for file
