# Supplementary material for: A new mutant genetic resource for tomato crop improvement by TILLING technology
Source: BMC Res Notes. 2010 Mar 12;3:69. doi: 10.1186/1756-0500-3-69 (PMC2845601; doi:10.1186/1756-0500-3-69)
Supplement: Additional file 1 — Nucleotide alignment. Additional data file 1 is a figure showing a comparison analysis of a tilled 240 bp region of Expansin1 gene. This analysis shows that the identified induced point mutations are not part of natural variability. [file 1756-0500-3-69-S1.DOC]

**Additional file 1- Nucleotide alignment**


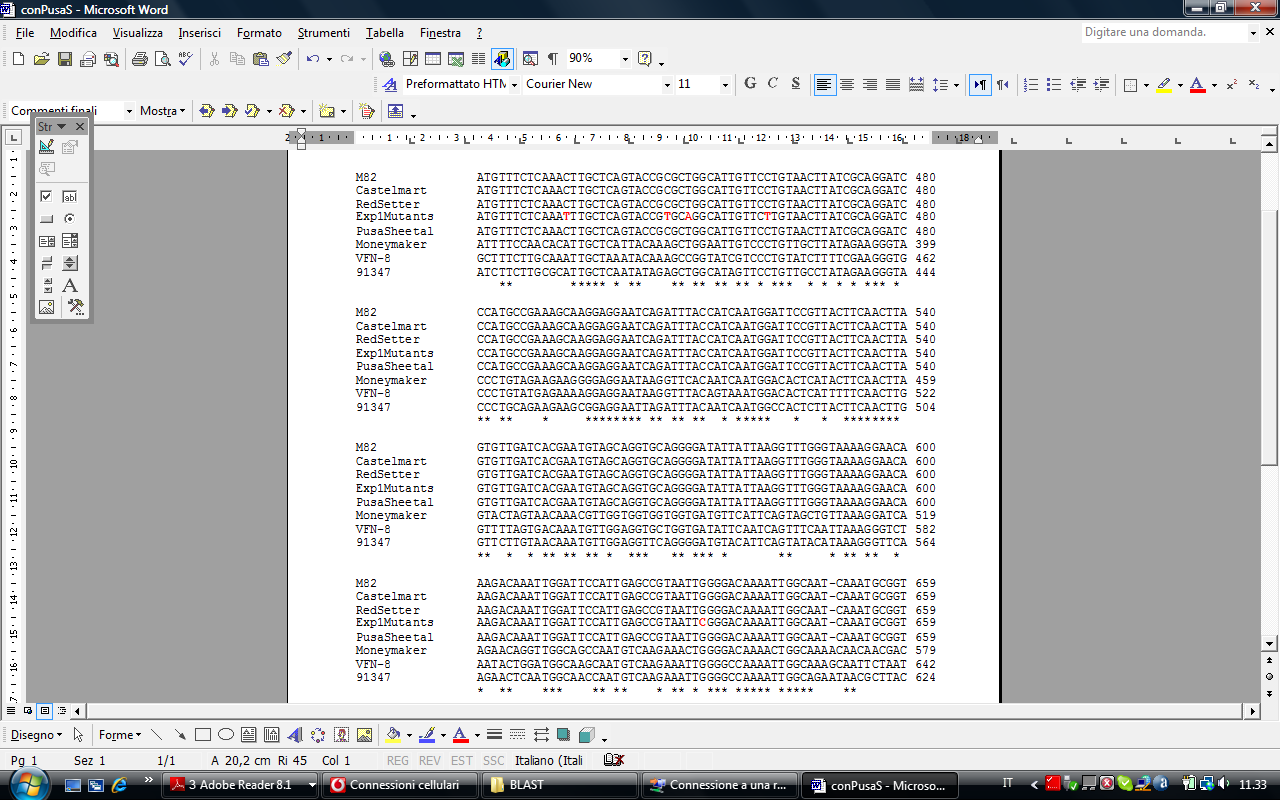


An example of comparison analysis of a tilled 240 bp region of *Expansin1* gene. The alignment includes seven sequences of *Solanum lycopersicon* cultivars: Red Setter and M82 (unpublished data), Castelmart [GenBank:U82123], Pusa Sheetal [GenBank:AF548376], VFN-8 [GenBank:AF096776], Moneymaker [GenBank:AJ560647], 91347 [GenBank:AF059489] and a mutant sequence (Exp1Mutants) containing five non-GC/AT changes identified, in this region, in five different tomato mutant lines through TILLING analysis. The discovered nucleotide mutations are marked in red; the arrowheads indicate the position of the nucleotide changes and the box indicates the position of the G/C transversion never found in other plant species by TILLING analysis.
